# Pattern recognition in lymphoid malignancies using CytoGPS and Mercator

**DOI:** 10.1186/s12859-021-03992-1

**Published:** 2021-03-01

**Authors:** Zachary B. Abrams, Dwayne G. Tally, Lin Zhang, Caitlin E. Coombes, Philip R. O. Payne, Lynne V. Abruzzo, Kevin R. Coombes

**Affiliations:** 1 Department of Biomedical Informatics, The Ohio State University, Columbus, OH 43210 USA; 2 The Center for Genomic Advocacy At Indiana State University, Terre Haute, IN 47809 USA; 3 Institute for Informatics, Washington University School of Medicine in St. Louis, St. Louis, MO 63108 USA; 4 Department of Pathology, The Ohio State University, Columbus, OH 43210 USA

**Keywords:** Karyotype, Pattern recognition, CytoGPS, Mercator, Lymphoid malignancies

## Abstract

**Background:**

There have been many recent breakthroughs in processing and analyzing large-scale data sets in biomedical informatics. For example, the CytoGPS algorithm has enabled the use of text-based karyotypes by transforming them into a binary model. However, such advances are accompanied by new problems of data sparsity, heterogeneity, and noisiness that are magnified by the large-scale multidimensional nature of the data. To address these problems, we developed the Mercator R package, which processes and visualizes binary biomedical data. We use Mercator to address biomedical questions of cytogenetic patterns relating to lymphoid hematologic malignancies, which include a broad set of leukemias and lymphomas. Karyotype data are one of the most common form of genetic data collected on lymphoid malignancies, because karyotyping is part of the standard of care in these cancers.

**Results:**

In this paper we combine the analytic power of CytoGPS and Mercator to perform a large-scale multidimensional pattern recognition study on 22,741 karyotype samples in 47 different hematologic malignancies obtained from the public Mitelman database.

**Conclusion:**

Our findings indicate that Mercator was able to identify both known and novel cytogenetic patterns across different lymphoid malignancies, furthering our understanding of the genetics of these diseases.

## Background

### Cytogenetics

As biology and medicine advance, our ability to generate ever-increasing amounts of data also expands [1]. While a boon for biomedical research, this increase in the volume and diversity of data poses challenges to data scientists [2]. Issues of noisiness, dimensionality, and heterogeneity can prove problematic when performing large-scale biomedical analyses [3]. These problems become more common and more severe as larger, higher-dimensional data sets are collected; as a result, some biomedical data remain underused.

For years, technical issues have limited the use of karyotype data in secondary computational analyses. Karyotype data are one of the most common forms of genetic information collected on patients, since cytogenetic karyotyping is part of the standard of care for most hematologic malignancies [4]. The current standard for large-scale cytogenetic analyses is manual classification by cytogenetic pathologists. This is extremely time consuming and can introduce human error into downstream analysis. Thus, these data have not been used in large-scale computational analyses because of the text-based standard format in which they are recorded [4]. In response, we recently developed the CytoGenetic Pattern Sleuth (CytoGPS), a tool that converts karyotypes into binary vectors that can be analyzed using modern computational methods [5]. However, CytoGPS is only a first step in understanding and exploring these data, since it merely enables (but does not carry out) the application of pattern recognition methods. To actually apply such methods systematically, we developed the Mercator R package. Mercator provides a consistent, unified interface to a suite of unsupervised pattern recognition algorithms. Mercator uses 10 distance metrics between binary vectors, selected from 76 metrics described and classified in a review paper by Choi et al. [6], to provide a representative sample of this wide scope of different metrics. Mercator also supports five data visualization methods designed for both standard and high dimensional data analysis; the visualization tools work with arbitrary distance metrics for any data type, not just binary. Mercator makes it easy to produce visualizations with consistent color schemes. More importantly, since cluster labels from different unsupervised algorithms are arbitrary, Mercator includes tools to synchronize and compare these labels. Thus, Mercator enables the exploratory unsupervised analysis of large, high-dimensional data sets, accompanied by clear, easy visualizations.

### Research design

In this article, we apply CytoGPS and Mercator to understand the structure of a data set containing more than 22,000 karyotypes from lymphoid malignancies. Lymphoid cells are one of the two most common cell types from which leukemias and lymphomas are derived, the other cell type being myeloid cells [7, 8]. Lymphoid cells include B cells, T cells, and natural killer (NK) cells. The current World Health Organization (WHO) classification of lymphoid malignancies [9] incorporates a variety of factors, including cytogenetics, cell-of-origin, location (bone marrow, blood, lymph node, etc.), clinical findings, immunophenotype, histological patterns (e.g., follicular or diffuse), and mutations or rearrangements of specific genes. The current WHO classification of lymphoid malignancies includes at least 60 subtypes; the historical karyotype data from the Mitelman. Database of Chromosome Aberrations and Gene Fusions in Cancer includes 47 named subtypes [10]. These subtypes include chronic lymphocytic leukemia (CLL), which is known to include at least four prognostic subgroups defined by different cytogenetic abnormalities [11]. Similarly, various cytogenetic abnormalities are known to be prognostic in subsets of acute lymphocytic leukemia (ALL) [12, 13]. One of the strengths of the Mercator approach is its ability to discover, visualize, and interpret large numbers of subtypes in large data sets. Here, our goal is to apply Mercator in order to determine whether lymphoid malignancies can be separated into clusters based on their patterns of cytogenetic abnormalities alone.

## Implementation

We first describe the data source, then the software packages and computational algorithms used to perform the analysis.

### Data

Cytogenetic data were obtained from the Mitelman Database of Chromosome Aberrations and Gene Fusions in Cancer, a curated, publicly available database containing all cytogenetic karyotypes published since the early 1970s [10]. When downloaded on 4 April 2019, it contained 22,741 karyotypes from patients with lymphoid malignancies, classified into 47 different disease domains. The karyotypes were written using the International System for Human Cytogenomic Nomenclature (ISCN) [14]. The ISCN is a semi-structured, semi-context-free grammar that produces a textual representation of the complete genetic information seen by the cytogeneticist evaluating the sample. We transformed the ISCN karyotypes representations into a binary representation based on a loss, gain, and fusion (LGF) biological model using CytoGPS (www.cytogps.org) [5].

### Algorithms

The Mercator package (version 0.10.0) facilitates the exploration of binary data sets. Mercator (1) removes redundant features, (2) calculates a variety of distance metrics, and (3) generates multiple visualizations using a consistent color scheme and interface. This approach is designed to help users more easily navigate through the process of data analysis and visualization for pattern recognition. Although the Mercator package implements multiple distance metrics (Jaccard, Sokal & Michener, Hamming, Russell-Rao, Pearson, Goodman & Kruskal, Manhattan, Canberra, Binary and Euclidean) between binary vectors [6], in this article we rely primarily on a metric derived from Jaccard similarity [15]. The Jaccard similarity index between two binary vectors is defined as *J* = *N*_11_/(*N*_11_ + *N*_10_ + *N*_01_), where *N*_*ij*_ is the number of entries where the first vector contains the value *i* and the second vector contains the value *j*. Because it ignores the “insignificant” 0–0 matches, it is particularly well adapted to finding structure in sparse binary data. (For comparison, the Additional files 1 and 2 also investigates the Soakl–Michener and Goodman–Kruskal metrics.)

Mercator relies on the Thresher (version 1.1.2) and PCDimension (version 1.1.11) R packages to remove outliers and to determine the number of clusters [16, 17]. The number of clusters depends on the number of significant principal components, which is determined using the Bayesian method of Auer and Gervini [18]. Next, samples are assigned to clusters using Partitioning Around Medoids (PAM) [19]. Although PAM is the default clustering algorithm, Mercator allows the user to apply their preferred clustering algorithm before using its visualization tools. Finally, Mercator provides an interface to data visualization (with a consistent color scheme) using a variety of techniques including multidimensional scaling (MDS) [20], hierarchical clustering [21], t-distributed Stochastic Neighbor Embedding (t-SNE) [22], and adjacency graphs. To simplify the visualization of graphs with more than 20,000 nodes, we also used Mercator to perform down-sampling. This approach was inspired by Peng Qiu’s implementation of the Spanning-tree Progression Analysis of Density-normalized Events (SPADE) clustering algorithm for mass cytometry data [23]. The main point is to under-sample the densest regions of the data space to make it more likely that rare clusters will still be adequately sampled. Mercator is available as an R package at (https://cran.r-project.org/web/packages/Mercator/index.html) where further information on the packages available.

## Results

In this section, we present results using the Jaccard distance metric. For theoretical reasons, we prefer the Jaccard metric, which is directly specifically designed to calculate distances for sparse binary data, where most of the values are zero [15]. Since most of each patient’s genome is normal, the cytogenetic data vectors for each patient are sparse. In the Mitelman data set, most cytogenetic features are also sparse, occurring in relatively small subsets of patients. However, analogs of all four figures and the final table from parallel analyses using the Sokal–Michener distance and the Goodman–Kruskal distance are presented in Additional files 1 and 2.

### Number of components and clusters

**Fig. 1:**
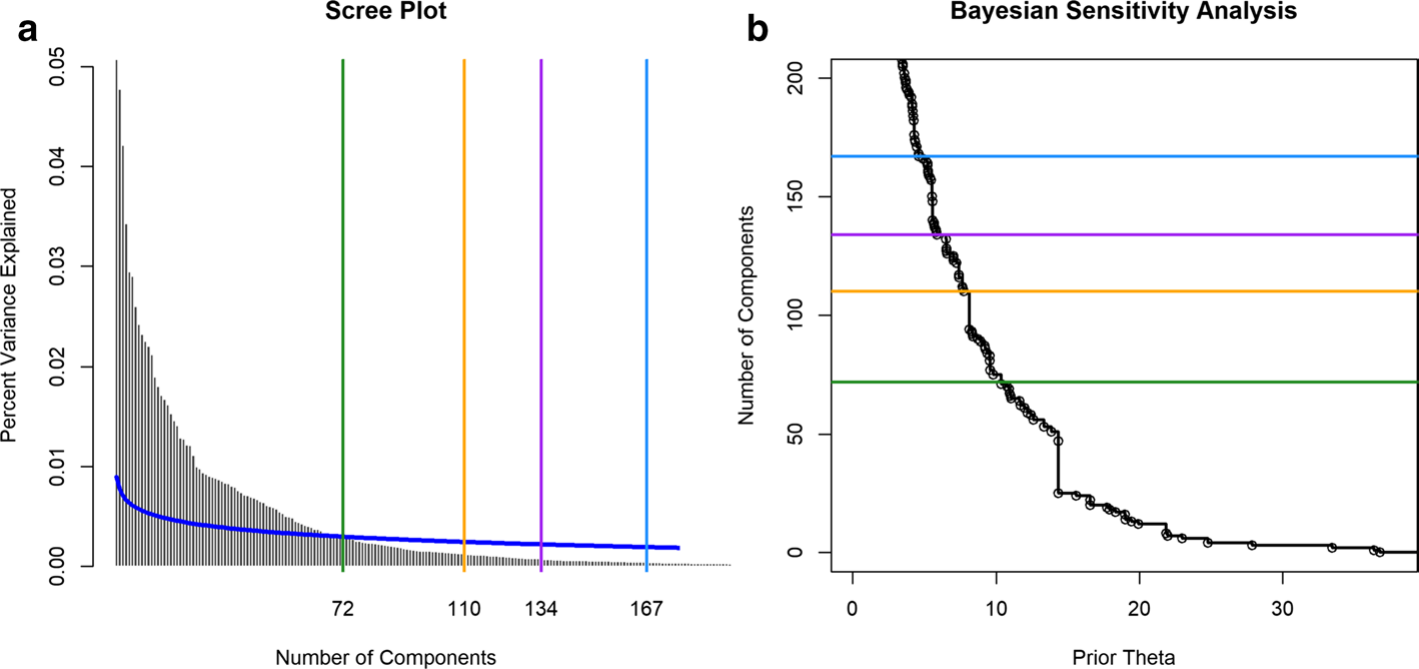
Number of principal components. **a** The scree plot shows the percent of variance explained. **b** The Auer–Gervini plot shows the maximum posterior number of components as a step function of the parameter theta selecting an exponentially decaying prior. In both panels, the green line is the number (N = 72) selected by the broken-stick model. The orange (N = 110), purple (N = 134), and blue (N = 167) lines mark “long” steps that are potential cutoffs for the number of components. We selected N = 134

We applied CytoGPS to the lymphoid malignancy samples from the Mitelman database, which generated a binary matrix of 22,741 samples and 2748 binary LGF features. Because large-scale copy number changes such as gains or losses of entire chromosomes affect many cytogenetic bands in the same way, we next removed any redundant features (i.e., features represented by identical vectors across the full data set). This step reduced the size to 1,144 unique features. We then applied Thresher to the features in order to identify “outliers” that do not contribute significantly to the principal components [16]. After this step, 814 unique informative features remained. Transposing the data, we also used the Jaccard metric to compute the distance between samples based on cytogenetic profiles. We used Thresher to find the number, K, of significant components (Fig. 1). We then assigned patient karyotypes to clusters using PAM with K = 134.

### Data visualizations

**Fig. 2:**
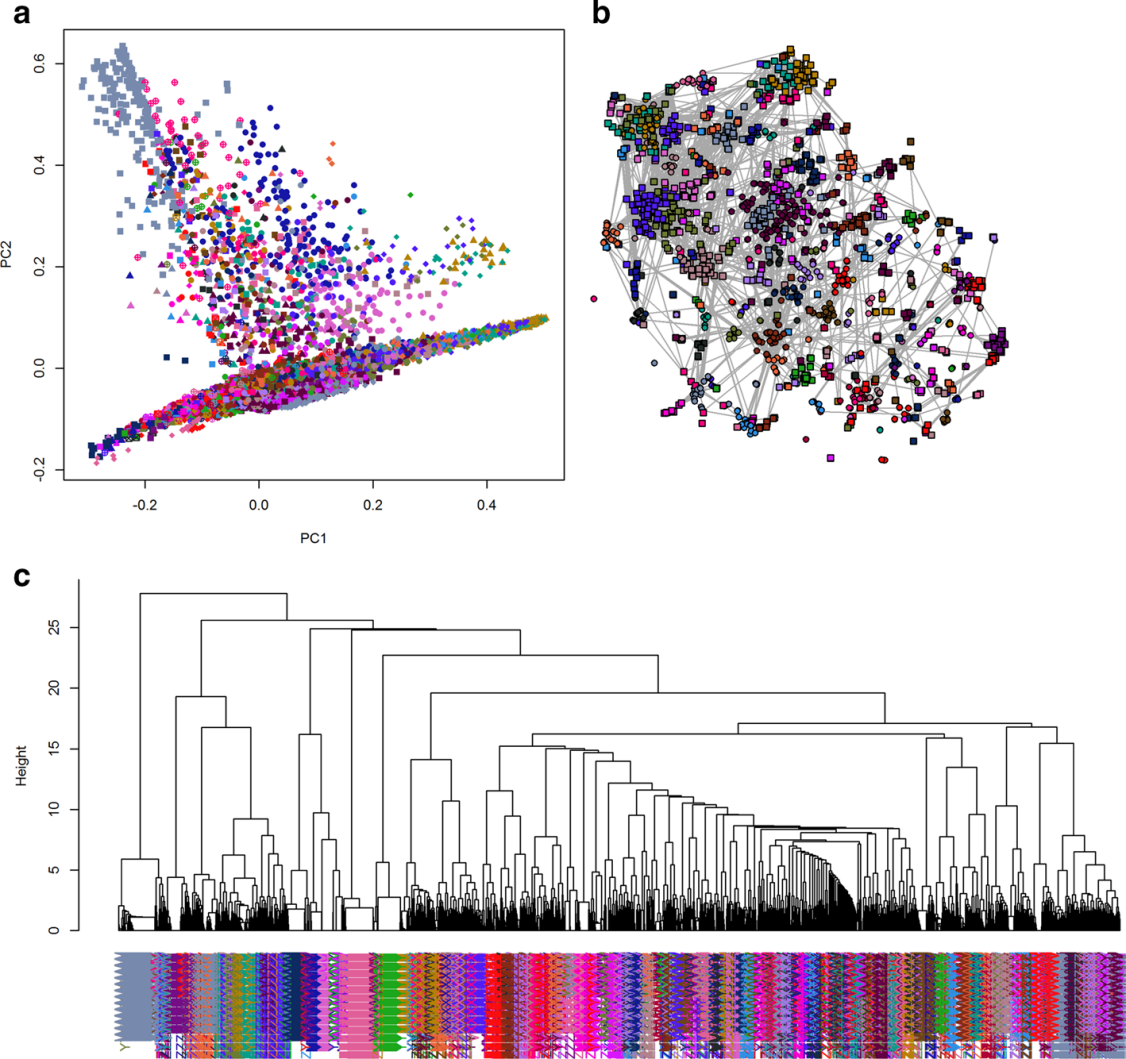
LGF binary karyotypes color-coded based on PAM clustering based on Jaccard distance were visualized using three methods. **a** Multi-dimensional scaling. **b** Down-sampled adjacency graph. **c** Hierarchical clustering using Ward’s linkage rule

**Fig. 3:**
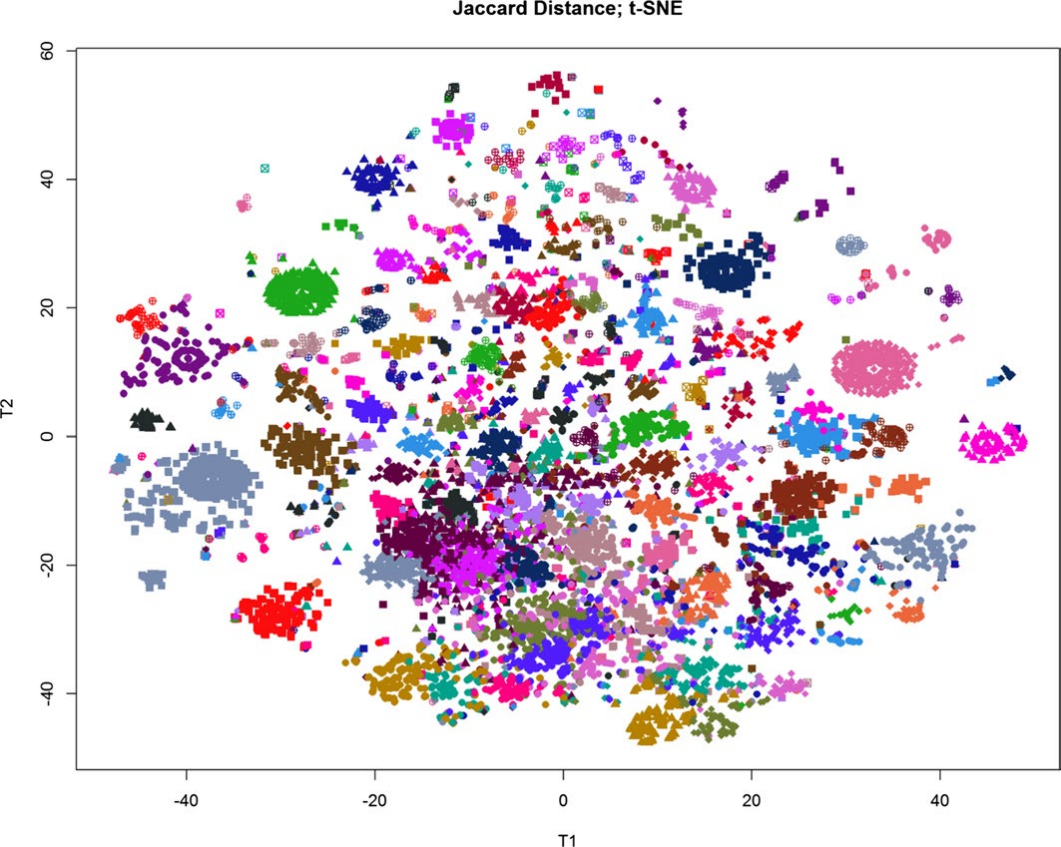
T-distributed Stochastic Neighbor Embedding (t-SNE) plot of the 134 karyotype clusters. Samples are color coded based on PAM clustering using Jaccard distance

To visualize the results of PAM clustering, we applied a variety of standard methods (Fig. 2). As an initial pass through the data, we performed hierarchical clustering using the Jaccard distance matrix. The first two principal coordinates derived from multi-dimensional scaling were unable to separate the PAM-defined clusters, which is unsurprising since we believe the principal component dimension to be K = 134 (Fig. 2a). In order to visualize the distance matrix as an adjacency graph, we down-sampled the data from 22,000 nodes to 2000 (Fig. 2b). Nodes were connected by an edge if the Jaccard distance was less than 0.6 or, equivalently, if the Jaccard similarity was greater than 0.4. This threshold was determined by identifying an inflection point in the distribution of all Jaccard values (data not shown). This threshold thus removed uninformative edges while preserving biologically informative connections. This would connect nodes if the two corresponding karyotypes shared 40% or more of their cytogenetic abnormalities, indicating a high degree a cytogenetics similarity. Both the adjacency graph and the dendrogram produced by hierarchical clustering (Fig. 2c) gave some visual support to the clusters found by PAM. Finally, we used the non-linear t-SNE algorithm to produce yet another plot of the data (Fig. 3). This visualization technique clearly shows separation between most of the PAM-clusters throughout the entire plot. Some of the tightest lusters form a distinguishing eye-shape; such clusters form an oval with a single point in the center. These clusters consist of groups of samples with identical or nearly identical karyotypes.

### Clustering and interpreting LGF features

**Fig. 4:**
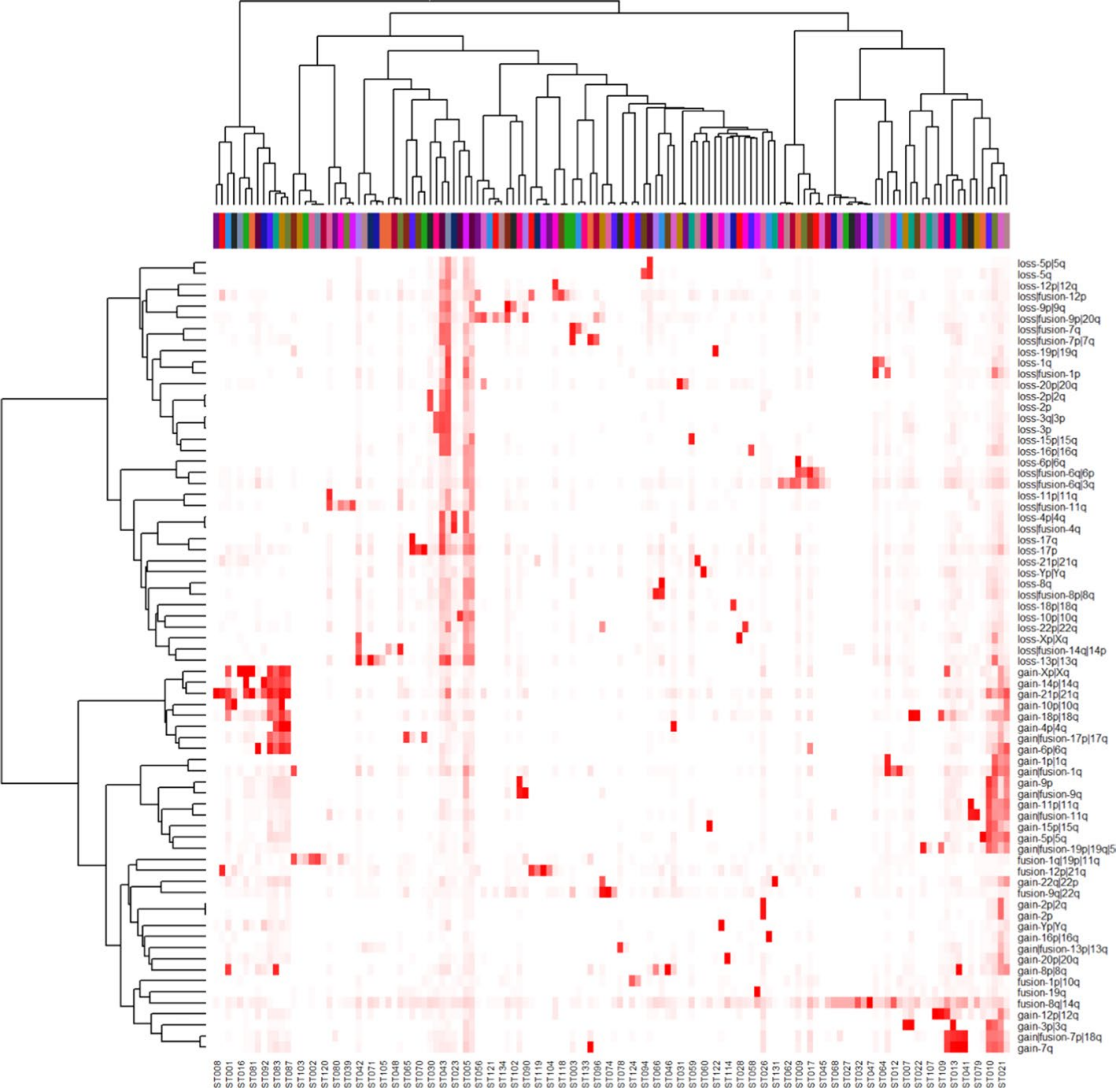
Heat map of high-frequency cytogenetic events (right) by clusters (bottom)

We now turned our attention to the 814 unique informative LGF features. Using Thresher and the Auer-Gervini method, we determined that the 814 features could be clustered into 72 groups. We again assigned features to clusters using PAM. In order to interpret these feature clusters, we determined all cytogenetic event types (loss, gain, or fusion) and chromosome bands present in any of the members of each cluster. (See the dendrogram and the labels along the side of Fig. 4.) Of the 72 clusters, 26 were only associated with a loss of a single chromosome or chromosome arm and 22 clusters were only associated with a gain of a single chromosome or chromosome arm. The remaining 24 clusters were associated with fusions and either one (N = 16 clusters) two (n = 7 clusters), or three (N = 1 cluster) chromosomes. Further, 11 of the 24 fusion-associated clusters were also associated with a loss of chromosomal material, 7 were associated with a gain, and 6 were pure fusions. All of the associations are consistent with single cytogenetic events.

### Interpreting sample clusters using high frequency cytogenetic aberrations

In order to interpret the sample-clusters, we next computed, for each cluster, the fraction of patients in that cluster who exhibited the well-characterized cytogenetic events defined by each of the 72 feature clusters described in the previous section. These frequency data were used to construct a two-way clustered heatmap based on Pearson correlation and Ward’s linkage (Fig. 4). The main split in the feature-dendrogram along the side of the heatmap is between losses (top branch) and gains and fusions (bottom branch). In other words, gains tend to occur along with other gains, and losses tend to occur along with other losses. Finally, we recorded the most frequent events (down to a frequency cutoff of 60%) in each sample-cluster. For 18 of the 134 clusters, at least one cytogenetic abnormality was present in at least 99% of the cases; for 42, at least one abnormality in at least 95% of case; for 50, 90%; for 71, 80%; for 84, 70%, and for 94, 60%. The 40 best characterized sample clusters by this measure are listed in Table 1. For each cluster we also calculated the disease prevalence based on karyotype disease labels. This was performed to with data interpretation, as some clusters contain multiple disease groups. The following diseases are part of at least one of the top 40 best characterized clusters: ALL = acute lymphocytic leukemia, CLL = chronic lymphocytic leukemia, Burkitt = Burkitt's lymphoma, FL = follicular lymphoma, DLBCL = diffuse large B-cell lymphoma and MM = multiple myeloma.

**Table 1:**
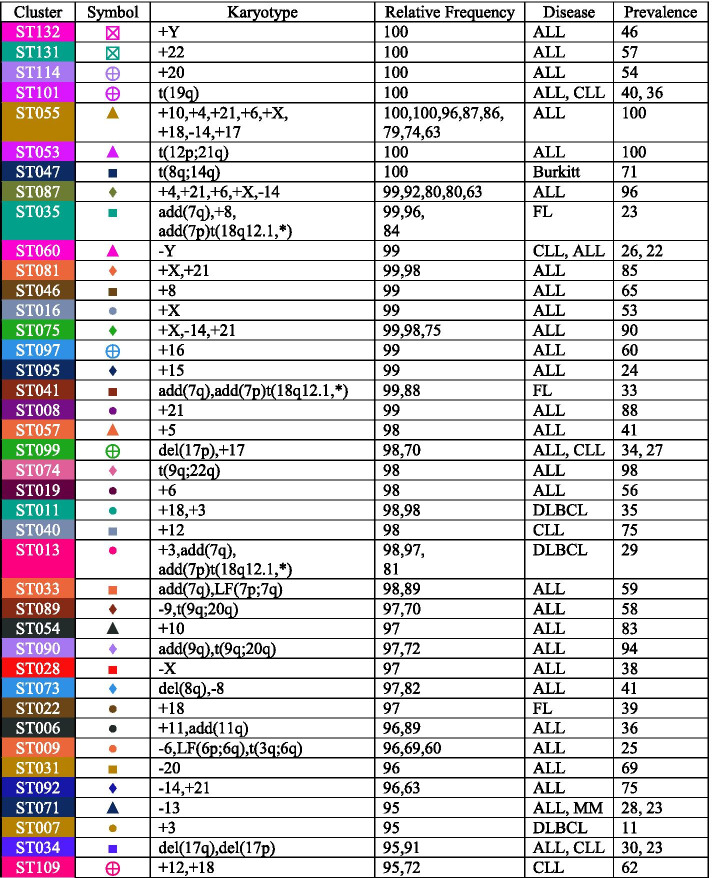
The top forty well characterized sample clusters

## Discussion

### Lymphoid karyotype clusters

We have shown that, by combining CytoGPS with Mercator to analyze 22,741 karyotypes obtained from the public Mitelman database, we are able to recover both simple and complex cytogenetic events that are important for understanding and classifying lymphoid malignancies. Using Mercator to cluster the binary LGF features, we found 72 clusters. Of these, 71 clusters represented simple losses restricted to one chromosome, simple gains restricted to one chromosome, or simple fusions involving at most two chromosomes. The remaining cluster was a fusion event involving three chromosomal arms: 1q, 11q, and 19p. Although more complicated than the others, this cluster represents a known phenomenon of “jumping” translocations involving 1q that has been seen in both lymphoid and myeloid malignancies [24, 25].

The lymphoid karyotypes from the Mitelman database represent 47 disease morphologies. Our analysis with Mercator found 134 clusters based on cytogenetics. We used the 72 elementary cytogenetic events above to characterize the 134 sample clusters. One of the well-known patterns is the t(8q;14q) translocation, which produces a fusion protein by juxtaposing the immunoglobulin heavy chain locus on chromosome 14 with the MYC oncogene on chromosome 8 [26]. This abnormality is the only recurrent event in cluster ST047 of Table 1, and occurs in 100% of the cases in that cluster. However, it is not unique to that cluster; as shown in Fig. 4, it is present at varying frequencies in the majority of lymphoid malignancy clusters. This finding can be explained by the fact that this translocation does not just occur as the sole abnormality in lymphoid malignancies, but also occurs in concert with many other combinations of abnormalities.

One of the strengths of using Mercator is its ability to uncover more complicated patterns that represent the recurrent co-occurrence of cytogenetic events. The most striking examples in Table 1 are clusters ST087 (with gains of four and loss of one chromosome) and ST055 (with gains of seven and loss of one chromosome). Both of these clusters display complex cytogenetic patterns that could only be uncovered using computational techniques and are unlikely to have been found simply by visual inspection of large sets of complex karyotypes. Looking deeper at these two clusters reveals that they share the events − 14 and + 21. In fact, these two events also co-occur in other clusters, including ST092 (which has only those two recurrent events) and ST075 (which combines them with an extra copy of the X chromosome). To our knowledge, this co-occurrence has not previously been recognized as a separate entity by cytogeneticists or hematopathologists. Preliminary visual inspections of the text-based karyotypes suggests that − 14 and + 21 almost always occur in the context of highly complex karyotypes where picking this pair out as a separate feature would be unlikely without computational assistance.

In general, the co-occurrence of a monosomy with a trisomy is unusual. A primary feature of Fig. 4 is that losses (monosomies) cluster together and gains (trisomies) cluster together, on separate branches in the (side) dendrogram. Hyperdiploidy (having more than the usual number of chromosomes) is a common feature of multiple myeloma [27] and of acute lymphoblastic leukemia [28] and has been reported in diffuse large B-cell lymphoma [29]. Hypodiploidy (having fewer than the normal number of chromosomes) is also common in lymphoid malignancies [30, 31].

A fundamental challenge when using any clustering method to perform unsupervised analysis arises from the difficulty of correctly ascertaining the number of clusters present in the data. We found that 94 (70%) of the 134 clusters have at least one cytogenetic abnormality that is present in at least 60% of the cases, and that many of those clusters have one or more abnormalities present at much higher frequencies. Thus, Mercator is able to identify high fidelity patterns and generates clusters that have a natural biological interpretation. It is possible, however, that the “true” number of clusters lies somewhere between the 134 found by Mercator and Thresher and the 47 known disease morphologies. Ideally, every cytogenetic cluster should be characterized by a unique combination of events.

### Distance metrics

We looked at different distance metrics to determine which metric would work best on cytogenetic data. In addition to the Jaccard distance, we performed our analyses using both the Sokal–Michener and Goodman–Kruskal metrics. These results are shown in Additional file 1 (Sokal–Michener) and Additional file 2 (Goodman–Kruskal). Sokal–Michener was not selected due to poor cluster differentiation (Additional file 1: Figure S2). Sokal–Michener did identify complex cytogenetic clusters, so it may be of research benefit for identifying recurrent complex events. Goodman–Kruskal identified weaker connections between karyotypes than Jaccard (Additional file 2: Figure S2), and thus was not selected. This is likely due to Goodman–Kruskal taking into consideration zero-zero matches when looking at binary data. This is in contrast to Jaccard, which only considers one–one matches to be meaningful. Since the LGF model data is a sparse binary vector it makes sense that a distance metric that only values one–one matches would outperform a metric that considers all matches.

One critically important aspect of Mercator is its use of shared cluster color schemes across different methods. It has been known for many years that humans are better than computers at determining visual patterns [32]. For this reason we designed Mercator to use a shared color scheme when using different methods on the same data set. This enables users to look at plots generated by different algorithms and visually compare them to determine the best algorithm for a given dataset. Keeping the color schemes consistent allows clusters that are based on a similar underlying characteristic can be compared across different clustering algorithms. This allows Mercator to leverage the intelligence of the researcher to help identify the best algorithm for a given dataset.

## Conclusions

In the future, it may be possible to address this issue by applying Mercator recursively. That is, we would first remove any cytogenetic event that is used to fully characterize one or more clusters at very high frequencies, and would then remove samples that only present with those abnormalities. We could then apply Mercator to the remaining features and samples to see if the resulting clusters can be characterized by other abnormalities at high frequency. We also intend to examine the associations between cytogenetically defined sample clusters and the known disease morphologies. A cursory examination suggests that the cytogenetic classification may be independent of and orthogonal to the known disease classification. If that observation holds up, then it will also be important to find other karyotype data sets that can be linked to clinical outcomes in order to test whether the cytogenetics can give better insight into an appropriate choice of therapies across disease types.

Mercator, in conjunction with CytoGPS, was able to identify biological patterns of shared elements within the cytogenetic profiles of different diseases. Data heterogeneity remains a very common problem in karyotype data analysis due to the innate linkage of cytogenetic features with one another due to colocating on the same chromosome. Mercator solves this problem by identifying unique feature sets and combining features to reduce the dimensionality while still preserving all relevant information. Mercator solves the related problem of data sparsity by selecting the proper measurement of distance. By utilizing the Jaccard distance, we were able to address the high levels of sparsity within our data set by focusing solely on 1 to 1 matches across our binary vectors. This elegant solution enabled both clustering and large-scale visualizations to be performed on an otherwise highly sparse and noisy high-dimensional data set.

Although we highlighted the usage of the Mercator package on binary cytogenetic data in this paper, it is important to note that Mercator is “data-type agnostic”. Many other forms of biomedical data could be easily processed and visualized using the Mercator methodology. This is particularly relevant in many omics fields where the large feature space requires clever feature reduction techniques, such as Thresher, to improve the overall computational analysis of the data. The standard visualizations used by Mercator will also aid these omics experiments, providing a clear visualization of the underlying data and thus a better understanding of the structure of omics data sets.

### Availability and requirements

- **Project name:** Mercator
- **Project home page:** https://CRAN.R-project.org/package=Mercator
- **Operating system(s):** Windows/macOS
- **Programming language:** R
- **Other requirements:** R (≥ 3.5), Thresher (≥ 1.1)
- **License:** Apache License (= = 2.0)
- **Any restrictions to use by non-academics:** No restrictions, only acknowledge authors contribution

## Supplementary Information

**Additional file 1.** Sokal Michener experiments.

**Additional file 2.** Goodman Kruskal experiments.

## Data Availability

All data generated or analyzed during this study are included in this published article, and its supplementary information files, or upon a reasonable request. The public data set Mitelman was used in this paper and can be found at: https://mitelmandatabase.isb-cgc.org/
