# Supplementary material for: Pattern recognition in lymphoid malignancies using CytoGPS and Mercator
Source: BMC Bioinformatics. 2021 Mar 1;22:100. doi: 10.1186/s12859-021-03992-1 (PMC7923511; doi:10.1186/s12859-021-03992-1)
Supplement: Supplementary file 1 — Additional file 1. Sokal Michener experiments. [file 12859_2021_3992_MOESM1_ESM.docx]

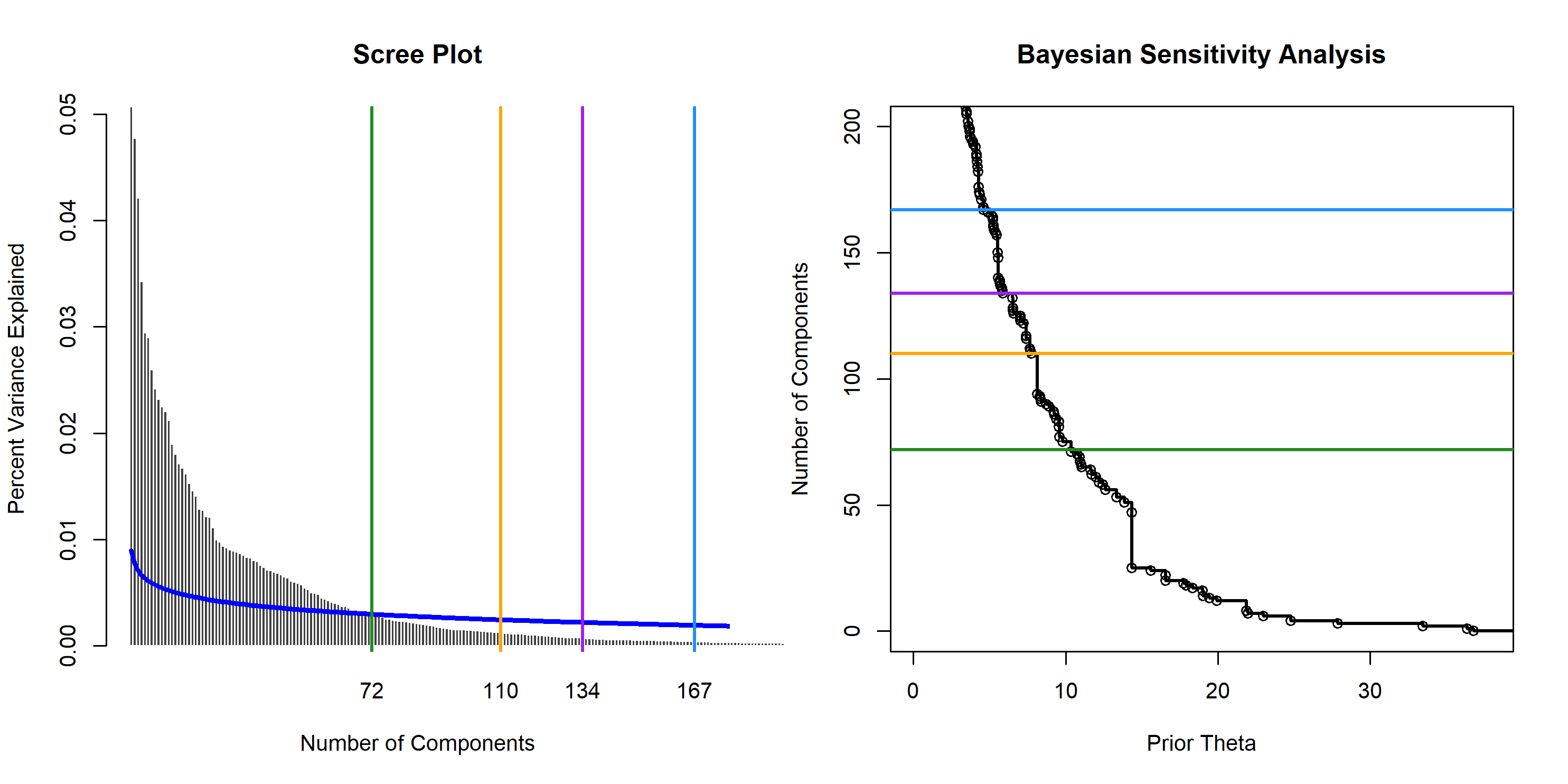


**Figure S1:** Number of principal components. (A) The scree plot shows the percent of variance explained. (B) The Auer-Gervini plot shows the maximum posterior number of components as a step function of the parameter theta selecting an exponentially decaying prior. In both panels, the green line is the number (N=72) selected by the broken-stick model. The orange (N=110), purple (N=134), and blue (N=167) lines mark “long” steps that are potential cutoffs for the number of components. We selected N=134. Because this step precedes the distance metric analyses the plots are the same as figure 1 in the main paper.


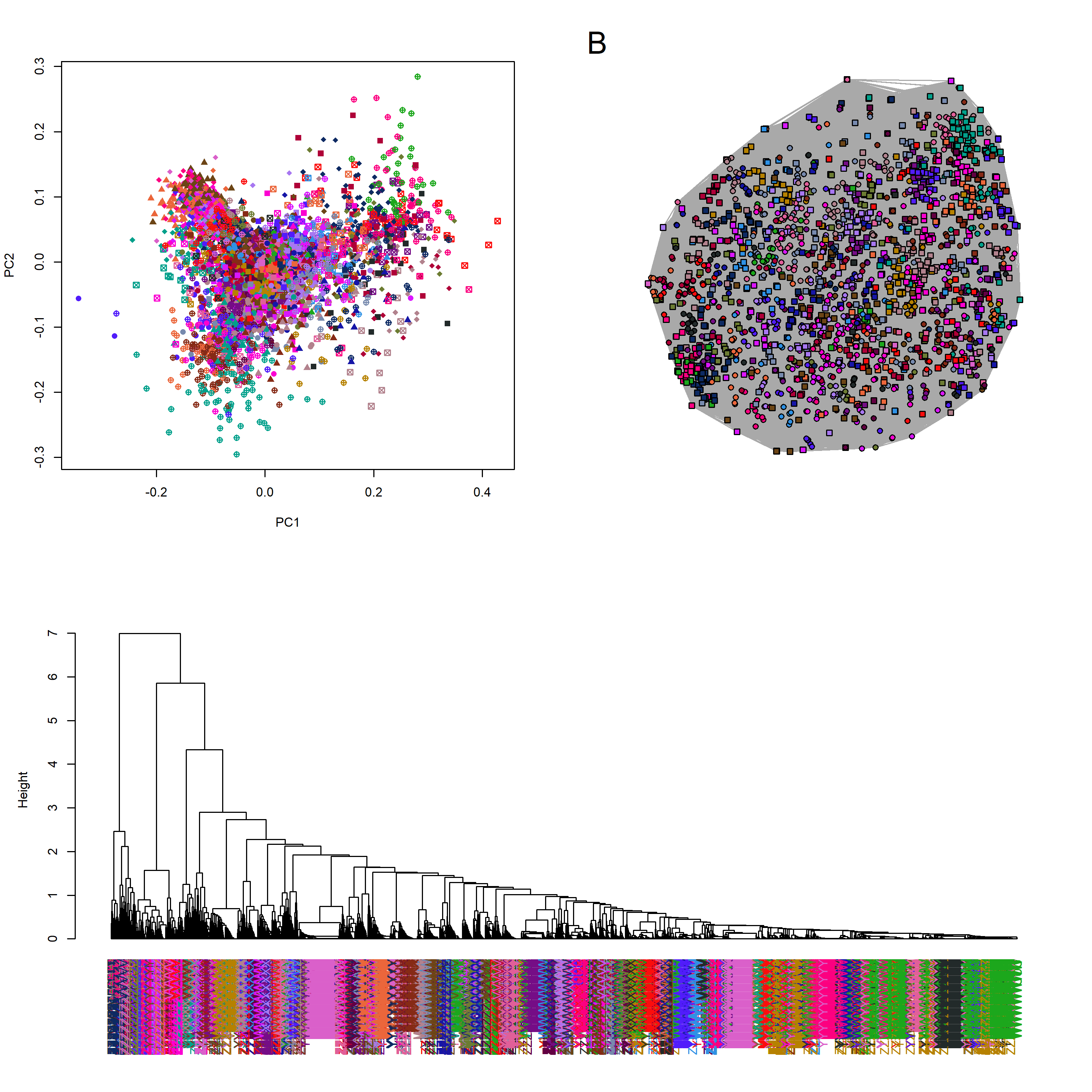


**Figure S2**: LGF binary karyotypes color-coded based on PAM clustering based on Sokal-Michener distance were visualized using three methods. (A) Multi-dimensional scaling. (B) Down-sampled adjacency graph. (C) Hierarchical clustering using Ward’s linkage rule. Overall there is worse cluster identity using Sokal-Michener than Jaccard, as seen in the down-sampling analysis.


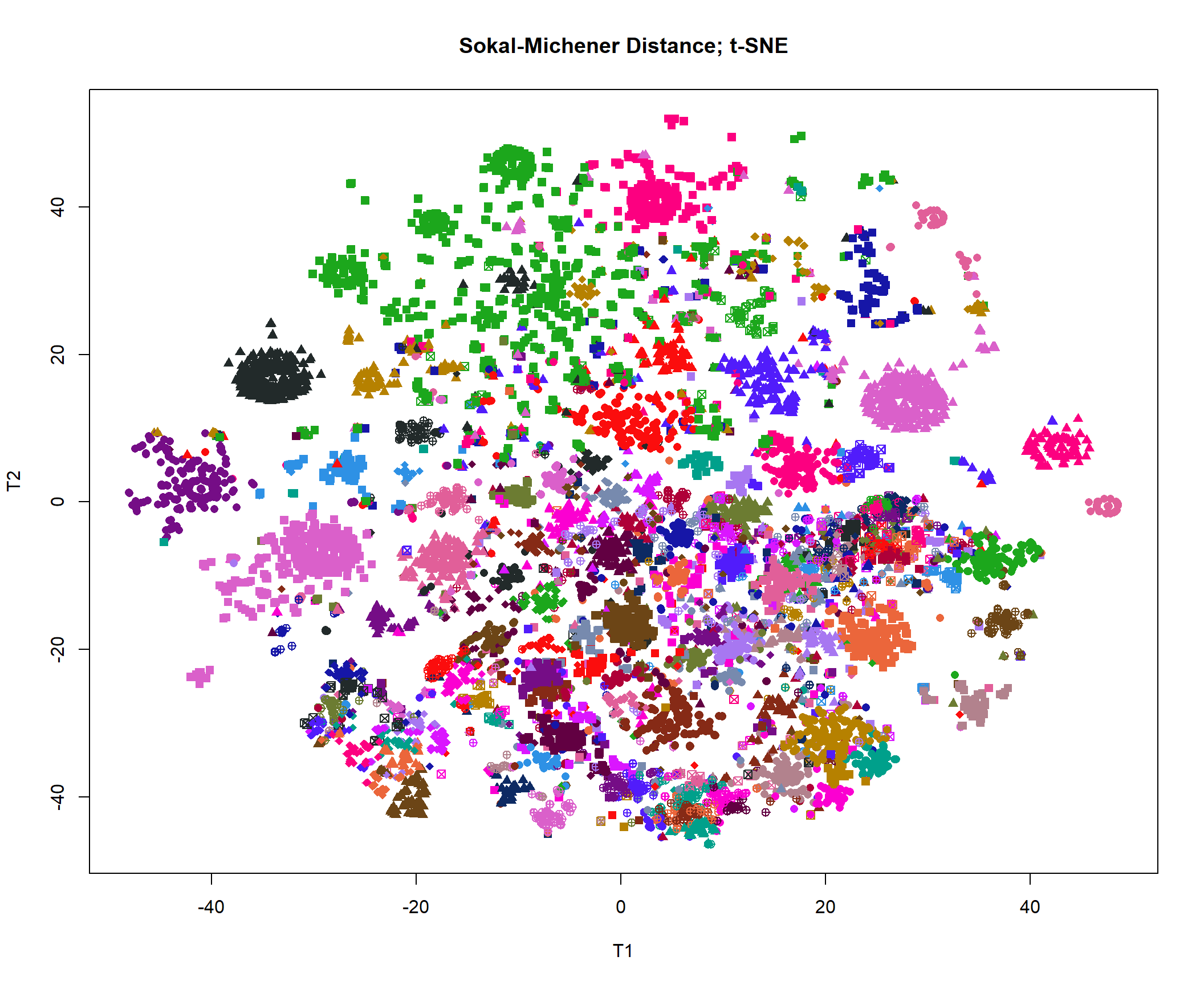


**Figure S3:** T-distributed Stochastic Neighbor Embedding (t-SNE) plot of the 134 karyotype clusters. Samples are color coded based on PAM clustering using Sokal-Michener distance. Some clusters appear to be visually distinct while others appear to be quite spread-out (Green squares). This may indicate a less than optimal cluster identification using Sokal-Michener distance.


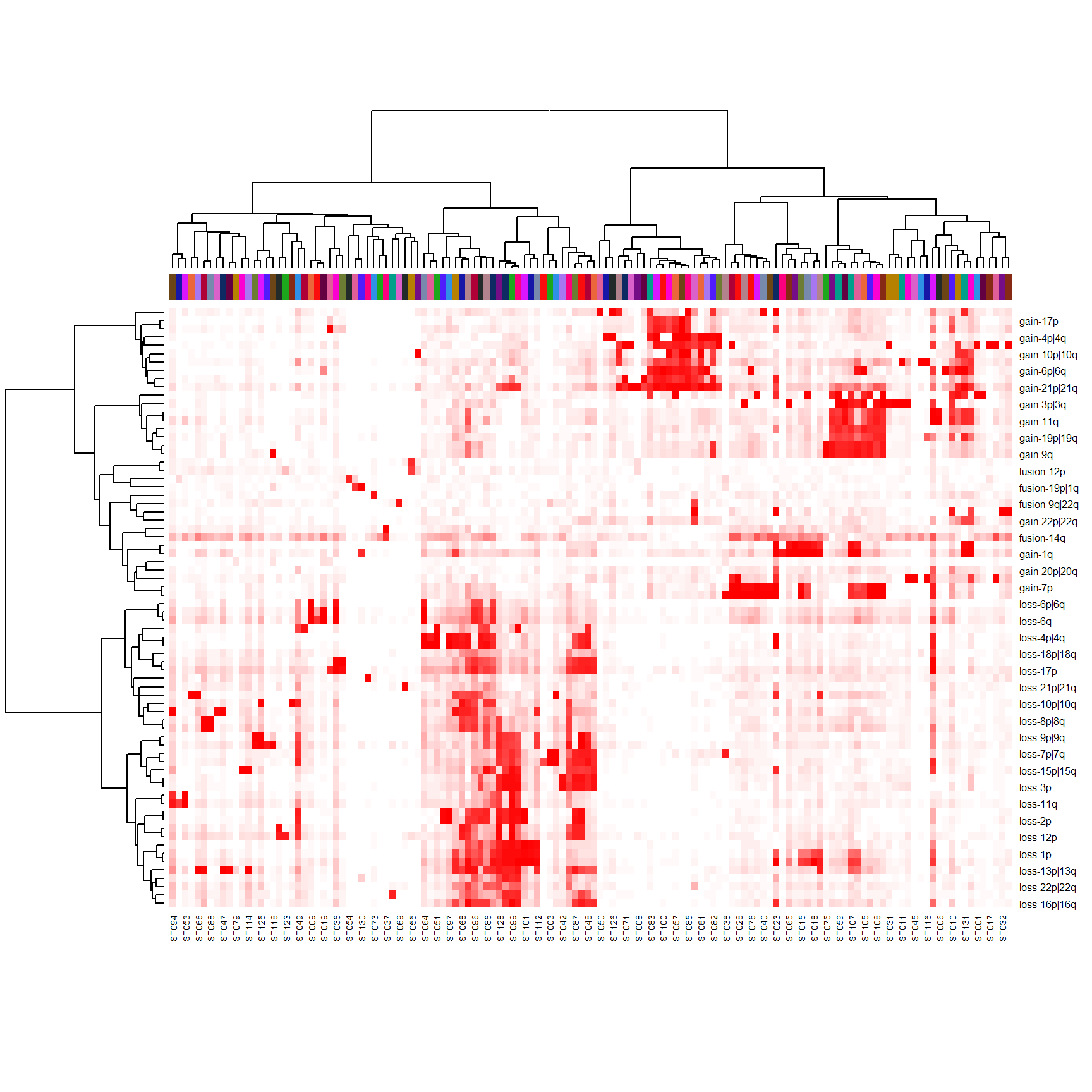


**Figure S4:** Heat map of high-frequency cytogenetic events (right) by clusters (bottom). The pattern of losses clustering with other losses and gains clustering with other gain present in the Jaccard analysis is also present when using Sokal-Michener. We also find more highly correlated cluster groups when using Sokal-Michener then when using either Jaccard of Goodman-Kruskal.

**Table S1:** The top forty well characterized sample clusters using Sokal-Michener distance. The characterizations tend to be much more complex than those from the Jaccard or Goodman-Kruskal distance metrics.

| Cluster | Symbol | Karyotype | Relative Frequency | Disease | Prevalence |
| --- | --- | --- | --- | --- | --- |
| ST047 | ■ | -14,-13 | 100,99 | MM | 59 |
| ST028 | ■ | +12,add(7q),add(7p), t(14q;*) | 101,100,100,  65 | FL, DLBCL | 34, 28 |
| ST132 | ⊠ | +1,add(1q),add(11q),+21,  +11,+18,+6,+12,+2,+22,  +10 | 100,100,81,81,  81,81,78,77,75,72,  69 | ALL | 69 |
| ST131 | ⊠ | +6,add(1q),+1,+21,-8,+15, +10,add(11q),+11,+5,+18, +22 | 100,99,96,96,86,86, 83,82,82,82,75,  73 | ALL | 96 |
| ST126 | ⊠ | +4,-8 | 100,98 | ALL | 61 |
| ST125 | ⊠ | -9,del(9p),-10 | 100,100,98 | ALL | 18 |
| ST124 | ⊠ | -9,del(9p),del(2p),-2,-3, del(3p),-7,-16,-17,del(7q), del(12p),del(17p),-12,-4, del(4q),-15,-20,-13 | 100,100,95,95,93,  91,89,89,86,86,  86,86,86,84,  84,77,75,73 | ALL | 89 |
| ST121 | ⊠ | -X,del(7q),-7 | 100,100,100 | ALL, MM | 40, 21 |
| ST116 | ⊕ | +12,+18,+15 | 100,100,64 | CLL | 67 |
| ST113 | ⊕ | add(7q),+3,+5,add(7p),  add(9q),add(9p),+15,+11,  add(11q),+15 | 100,99,99,99,  92,92,87,84,  83,83 | MM | 92 |
| ST111 | ⊕ | +4,+X,+21 | 100,100,82 | ALL | 93 |
| ST109 | ⊕ | del(1p),-5,del(3p),del(1p),  -2,del(2p),-3,-13,del(1q),  -15,-16,-19,-20,+21,  del(11q),-11,-7,del(9p),  -9,del(7q),-22 | 100,98,95,95,  94,94,94,94,92,  90,88,86,84,80,  78,77,71,70,  68,66,63 | ALL | 92 |
| ST108 | ⊕ | add(7q),add(7p),+3,  add(11q),+11,+15,+15,  add(9q),add(9p),+21 | 100,100,94,  88,87,85,83,  79,78,66 | MM | 87 |
| ST107 | ⊕ | add(1q),+1,+3,add(9q),  add(9p),+15,+5,add(7q),  add(7p),del(1p),+15,  add(11q),+11,-13 | 100,96,92,90,  87,84,84,82,  82,78,77,  74,72,64 | MM | 85 |
| ST106 | ⊕ | add(7q),add(7p),+5,  add(9q),add(9p),+15,  add(11q),+11,+15 | 100,100,94,  92,90,82,  81,79,76 | MM | 89 |
| ST105 | ⊕ | +6,+3,+15,add(9q),add(9p),  +5,add(7q),add(7p),+15,  +11 | 100,100,88,86,86,  84,77,77,75,  60 | MM | 98 |
| ST101 | ⊕ | del(2p),-2,del(1q),del(1p), del(1p) | 100,100,100,99,  98 | DLBCL, ALL | 37, 27 |
| ST099 | ⊕ | del(12p),del(11q),-12,-16,  -11,del(1p),del(2p),-15,  del(1p),del(3p),-3,-2,  del(1q),-13,-7,+21,del(7q),  -20,-19,-9,del(9p),-22 | 100,98,98,98,  95,95,95,95,  95,95,95,95,  95,88,84,82,82,  82,82,77,75,61 | ALL | 100 |
| ST097 | ⊕ | del(2p),del(4q),-2,-4 | 100,100,100,98 | DLBCL | 17 |
| ST093 | ♦ | +2,+21,+14,+6,+4,+X,  +22,t(92;22q) | 100,90,82,79,79,77,  76,75 | ALL | 90 |
| ST090 | ♦ | +6,+4,+21,+X | 100,100,93,79 | ALL | 99 |
| ST088 | ♦ | del(9p),-8 | 100,99 | ALL | 45 |
| ST085 | ♦ | -8,+10,+4,+21,+14,  +6,+X,add(17q),add(17p),  +18 | 100,100,100,96,94,  92,84,82,81,  80 | ALL | 100 |
| ST081 | ♦ | +10,+21,+4,+6,+X,  +18,+14 | 100,100,100,94,89,81,73 | ALL | 100 |
| ST080 | ♦ | +1,add(1q) | 100,100 | ALL | 29 |
| ST077 | ♦ | +4,+21,+6,+X,add(17q),  +14,add(17p),+18 | 100,98,93,92,90,  89,84,77 | ALL | 100 |
| ST074 | ♦ | +X,+12,t(14q;*) | 100,97,69 | FL, DLBCL | 44, 32 |
| ST072 | ▲ | -8,del(9p),-13 | 100,100,98 | MM | 47 |
| ST071 | ▲ | +X,+14,+21 | 100,99,83 | ALL | 90 |
| ST070 | ▲ | add(17q),+10,+4,add(17p),+21,+6,+X,+18,+14 | 100,100,100,99,  97,93,91,88,83 | ALL | 99 |
| ST066 | ▲ | -X,-13 | 100,99 | MM | 69 |
| ST065 | ▲ | add(1q),+3,+1 | 100,100,93 | MM | 28 |
| ST064 | ▲ | del(6q),-4,del(6q),del(4q),  -6 | 100,100,100,100,  92 | DLBCL | 21 |
| ST059 | ▲ | +3,+5,add(9q),+15,add(9p),add(11q),+11,+15 | 100,99,87,86,85,  80,76,76 | MM | 93 |
| ST057 | ▲ | +4,+5,+21,+6,+X,add(17q),add(17p),+14,+10,+18 | 100,99,97,92,89,88,82,79,78,71 | ALL | 100 |
| ST053 | ▲ | del(11q),-11 | 100,99 | ALL | 40 |
| ST051 | ▲ | -4,del(4q),-5 | 100,100,97 | ALL | 27 |
| ST037 | ■ | t(8q;*),t(14q;*) | 100,80 | Burkitt | 62 |
| ST036 | ■ | del(6q),del(6q),del(17p),  -17,-6 | 100,100,100,99,  92 | DLBCL | 22 |
| ST029 | ■ | add(7q),+5,add(7p),t(14q;*) | 100,99,99,66 | FL, DLBCL | 36, 25 |
