# Supplementary material for: Pattern recognition in lymphoid malignancies using CytoGPS and Mercator
Source: BMC Bioinformatics. 2021 Mar 1;22:100. doi: 10.1186/s12859-021-03992-1 (PMC7923511; doi:10.1186/s12859-021-03992-1)

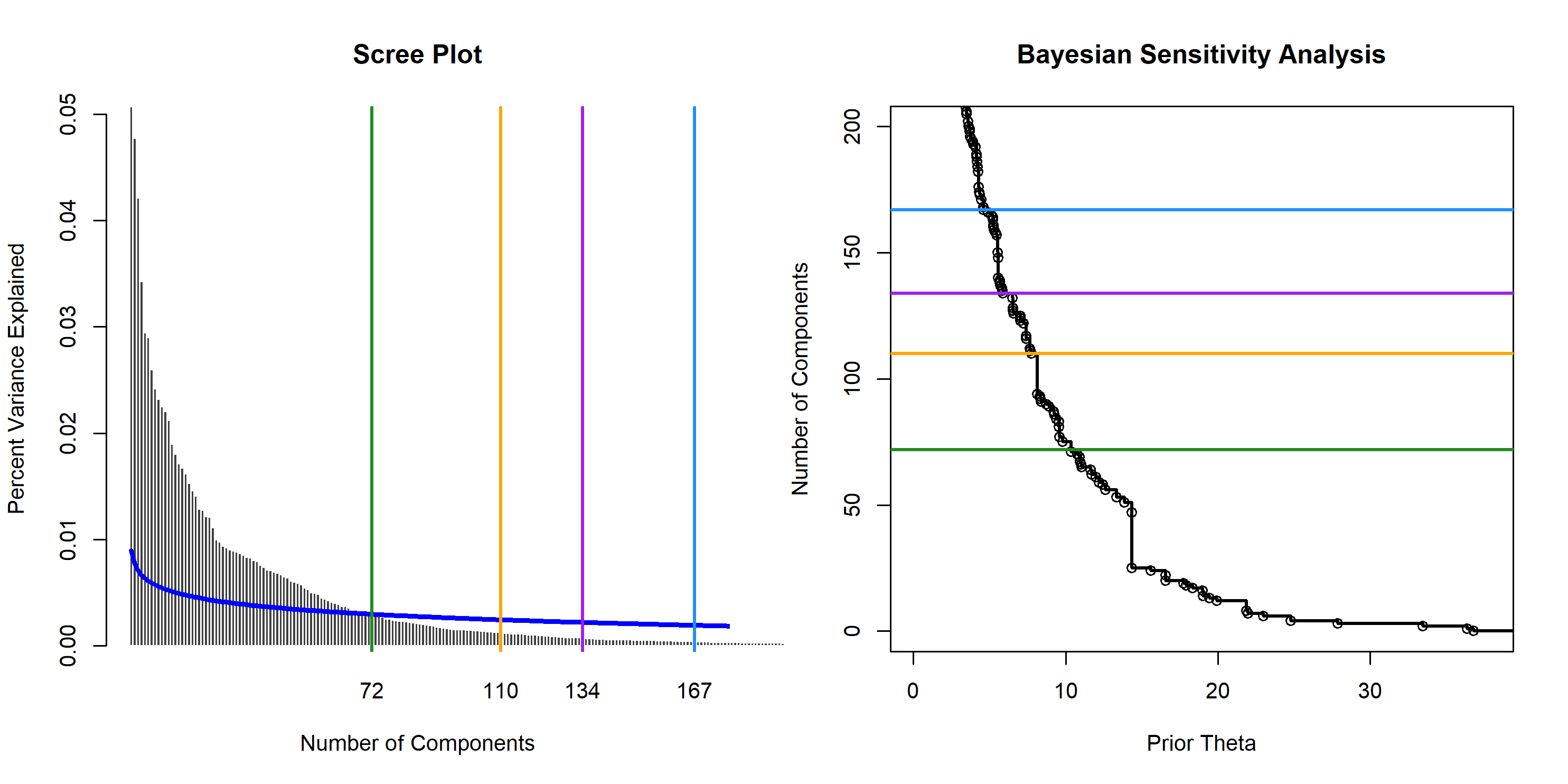


**Figure S1:** Number of principal components. (A) The scree plot shows the percent of variance explained. (B) The Auer-Gervini plot shows the maximum posterior number of components as a step function of the parameter theta selecting an exponentially decaying prior. In both panels, the green line is the number (N=72) selected by the broken-stick model. The orange (N=110), purple (N=134), and blue (N=167) lines mark “long” steps that are potential cutoffs for the number of components. We selected N=134. Because this step proceeds the distance metric analyses the plots are the same as figure 1 in the main paper.


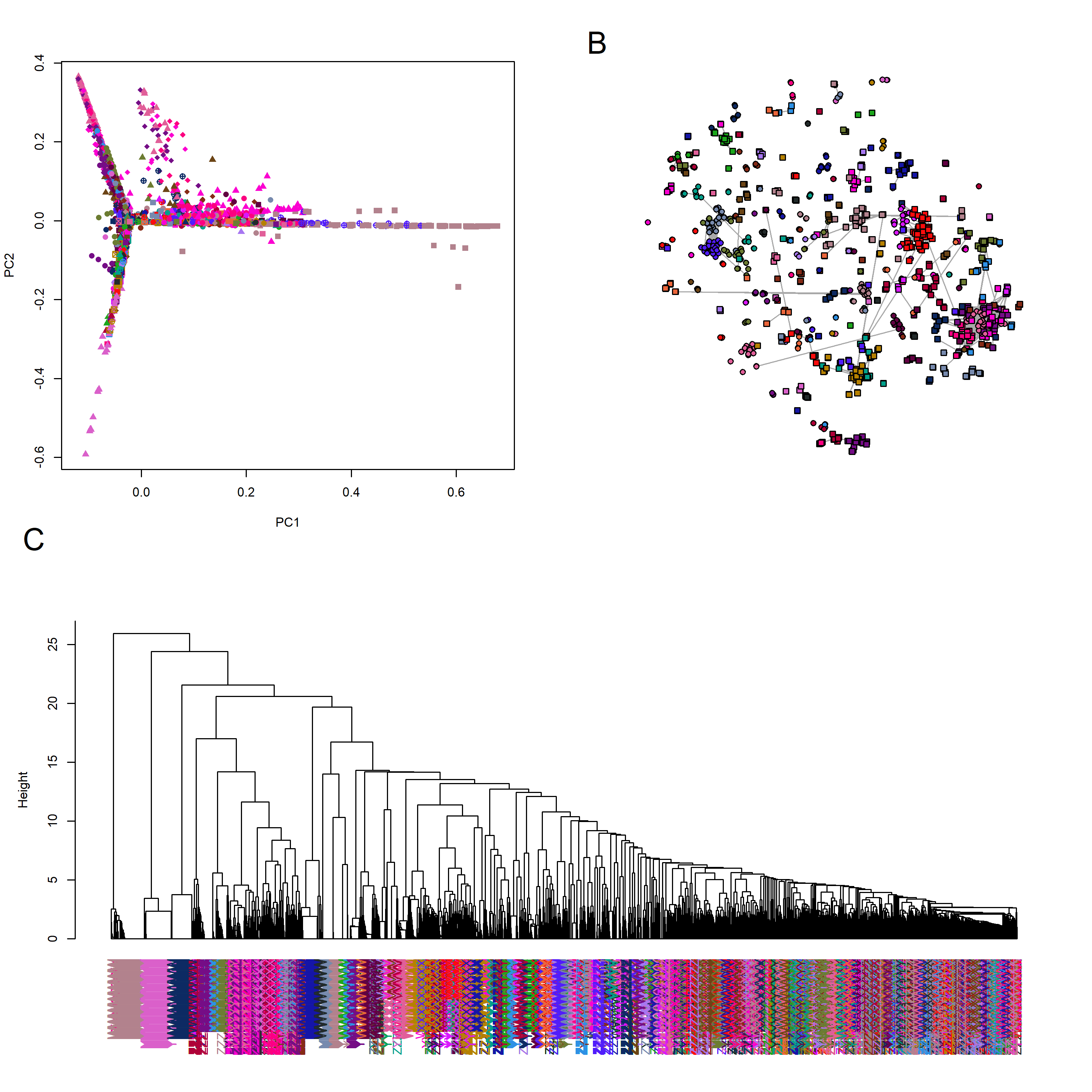


**Figure S2:** LGF binary karyotypes color-coded based on PAM clustering based on Goodman-Kruskal distance were visualized using three methods. (A) Multi-dimensional scaling. (B) Down-sampled adjacency graph. (C) Hierarchical clustering using Ward’s linkage rule. The down-sampling results show fewer connections then were seen in the Jaccard down-sampling analysis.


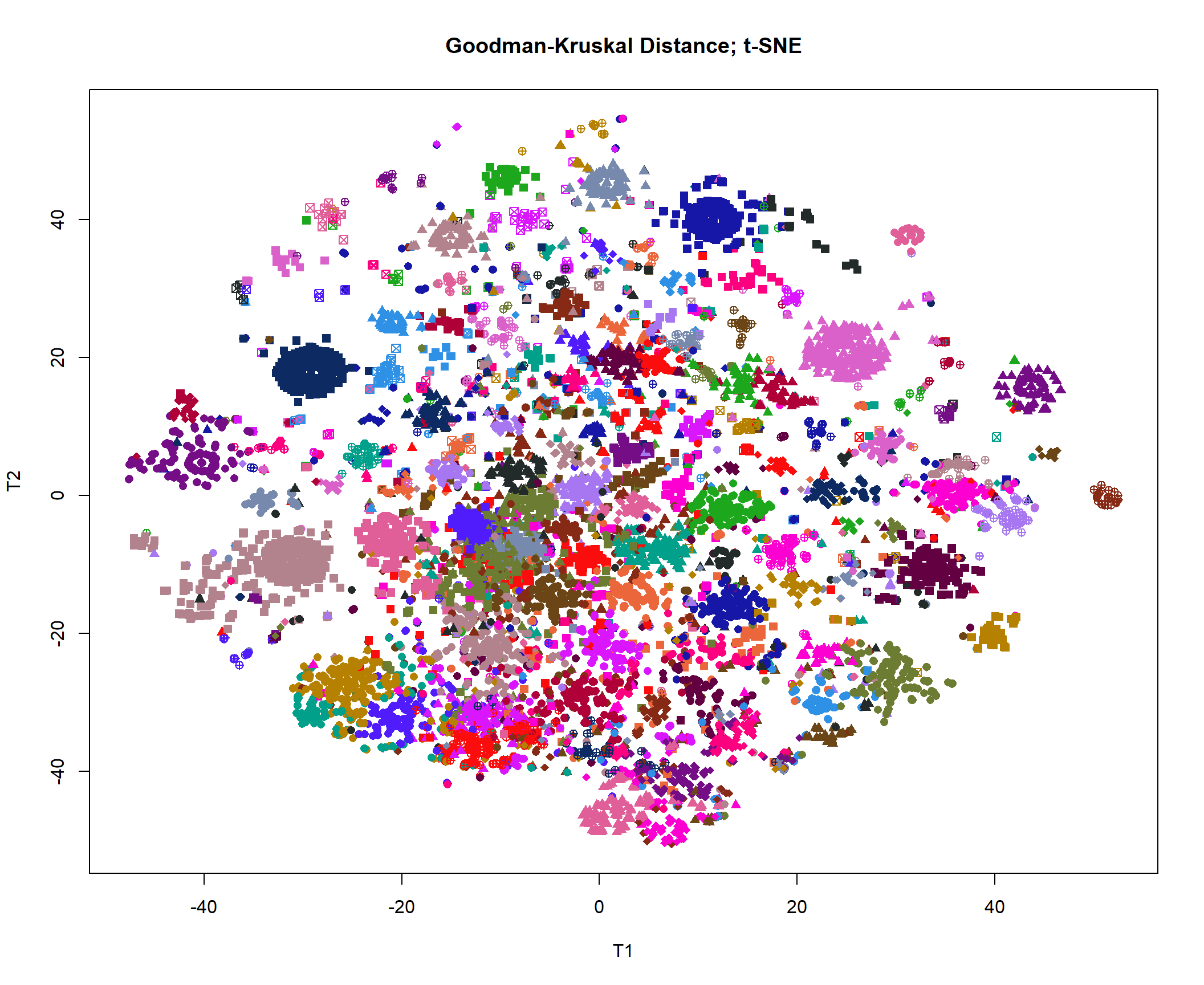


**Figure S3:** T-distributed Stochastic Neighbor Embedding (t-SNE) plot of the 134 karyotype clusters. Samples are color coded based on PAM clustering using Goodman-Kruskal distance. Goodman-Kruskal appears similar to Jaccard when compared visually.


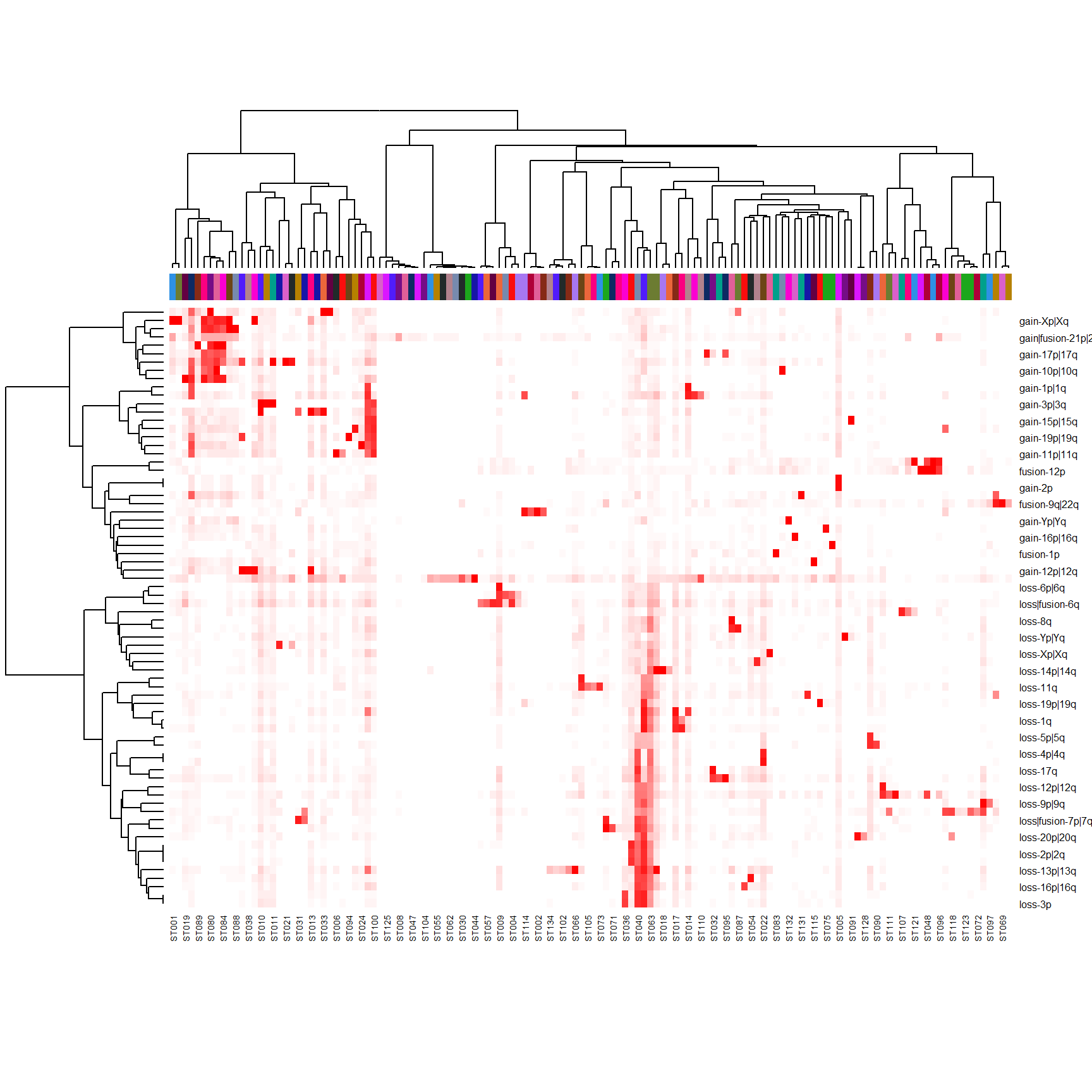


**Figure S4:** Heat map of high-frequency cytogenetic events (right) by clusters (bottom). The pattern of losses clustering with other losses and gains clustering with other gain present in the Jaccard analysis is also present when using Goodman-Kruskal. The cluster correlation patterns appear similar to those found using Jaccard.

**Table S1:** The top forty well characterized sample clusters using Goodman-Kruskal distance.

| Cluster | Symbol | Karyotype | Relative Frequency | Disease | Prevalence |
| --- | --- | --- | --- | --- | --- |
| ST106 | ⊕ | +12,+19,+18 | 101,78,76 | CLL | 70 |
| ST132 | ⊠ | +Y | 100 | ALL | 46 |
| ST115 | ⊕ | +20 | 100 | ALL | 53 |
| ST101 | ⊕ | t(12p;*) | 100 | ALL | 87 |
| ST099 | ⊕ | t(*;19q) | 100 | ALL, CLL | 37, 33 |
| ST094 | ♦ | +19 | 100 | ALL | 67 |
| ST080 | ♦ | +8,+14,+4,+X,-6,+18, +10,+17 | 100,85,85,78,70,69,69,63 | ALL | 99 |
| ST070 | ▲ | +X,+14 | 100,99 | ALL | 90 |
| ST060 | ▲ | +X,+12 | 100,98 | FL, DLBCL | 44, 29 |
| ST050 | ▲ | +10,+4,-6,+X,+18,  +14,+17 | 100,100,90,88,81, 76,71 | ALL | 100 |
| ST049 | ▲ | t(12p;*),t(*;21q) | 100,100 | ALL | 100 |
| ST048 | ■ | t(12p;*),t(*;21q),del(12p) | 100,74,74 | ALL | 91 |
| ST044 | ■ | t(8q;14q) | 100 | Burkitt | 71 |
| ST013 | ● | +12,+7 | 100,99 | FL, DLBCL | 34, 33 |
| ST010 | ● | +3,+7 | 100,99 | DLBCL, MM | 27, 20 |
| ST084 | ♦ | +4,-6,+X,+14,+17 | 100,88,85,67,66 | ALL | 98 |
| ST043 | ■ | +8 | 100 | ALL | 65 |
| ST024 | ● | +5 | 100 | ALL | 36 |
| ST001 | ● | +X | 100 | ALL | 83 |
| ST015 | ● | +X | 99 | ALL | 53 |
| ST056 | ▲ | -Y | 99 | CLL | 26 |
| ST039 | ■ | -14,-13 | 99,97 | MM | 55 |
| ST011 | ● | +3,+18 | 99,99 | DLBCL | 33 |
| ST091 | ♦ | +15 | 99 | ALL | 22 |
| ST019 | ● | -6 | 99 | ALL | 47 |
| ST021 | ● | +18 | 99 | FL, ALL | 23, 23 |
| ST033 | ■ | +8,+7 | 99,97 | FL | 19 |
| ST018 | ● | -14 | 99 | ALL | 47 |
| ST093 | ♦ | +16 | 99 | ALL | 58 |
| ST074 | ♦ | del(8q),LF(8) | 99,86 | ALL | 36 |
| ST006 | ● | +11 | 99 | ALL | 31 |
| ST038 | ■ | +12 | 99 | CLL | 75 |
| ST059 | ▲ | -9,t(9p;20q)* | 99,72 | ALL | 50 |
| ST083 | ♦ | t(1p;*) | 99 | ALL, FL | 33, 29 |
| ST095 | ♦ | LF(17),+17 | 98,69 | ALL, CLL | 35, 26 |
| ST016 | ● | +10 | 98 | ALL | 84 |
| ST007 | ● | +3 | 98 | DLBCL | 13 |
| ST069 | ▲ | t(9q;22q) | 98 | ALL | 98 |
| ST131 | ⊠ | +22 | 98 | ALL | 54 |
| ST089 | ♦ | +4 | 98 | ALL | 65 |


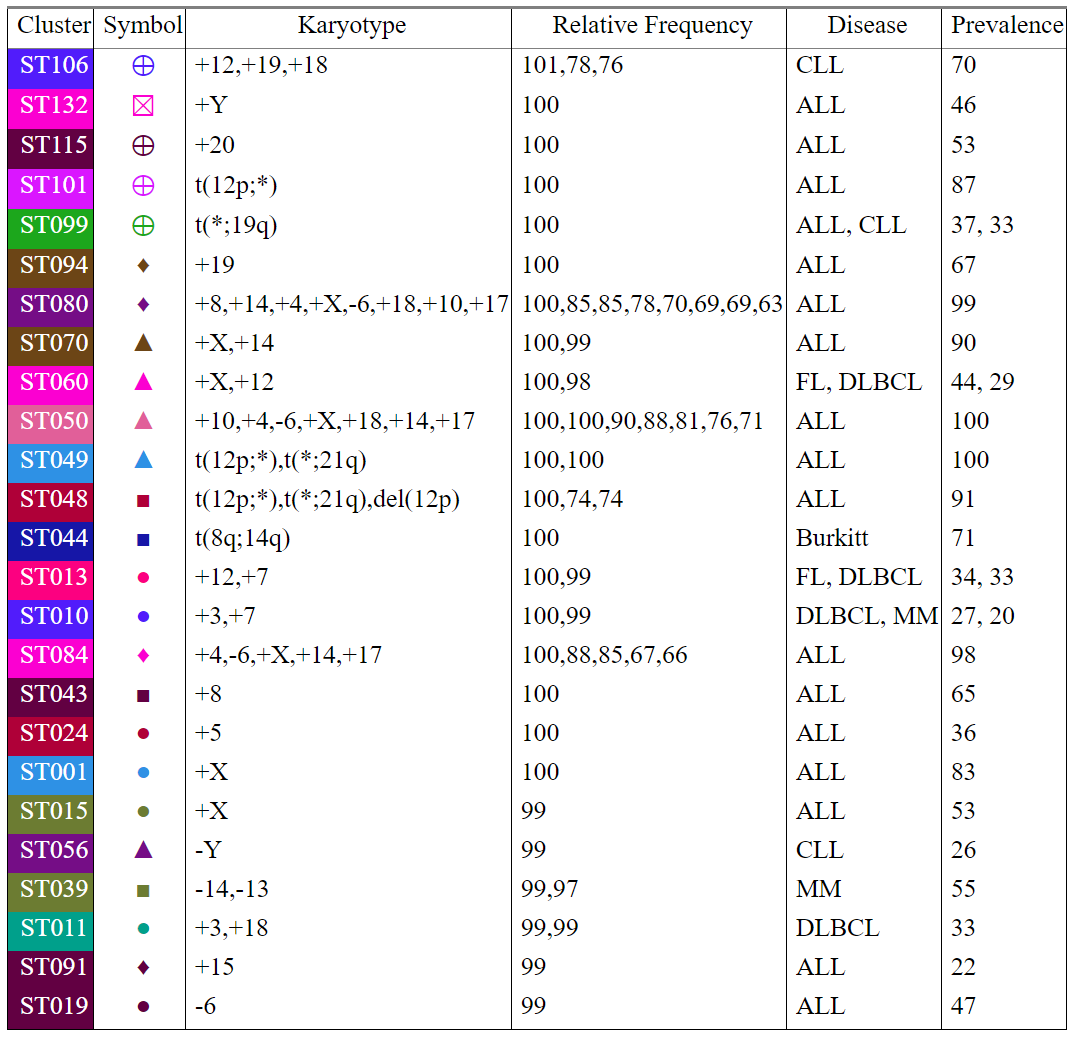

Supplement: Supplementary file 2 — Additional file 2. Goodman Kruskal experiments. [file 12859_2021_3992_MOESM2_ESM.docx]
